# From myth to bedside: a scoping review of the applications of the chimeric antigen receptor in rheumatology

**DOI:** 10.1007/s10238-025-01717-9

**Published:** 2025-06-06

**Authors:** Diego Niño-Torres, Gerardo Quintana-López, Gustavo Salguero

**Affiliations:** 1 Department of Internal Medicine, School of Medicine, Universidad Nacional de Colombia, Bogota, Colombia; 2 Department of Internal Medicine, School of Medicine, Universidad de Los Andes, Bogota, Colombia; 3 Rheumatology Section, Department of Internal Medicine, Fundación Santa fe de Bogotá University Hospital, Bogota, Colombia; 4 Advanced Therapies Unit, Instituto Distrital de Ciencia Biotecnología e Innovación en Salud-IDCBIS, 111611 Bogota, Colombia; 5 Present Address: Departamento de Medicina Interna, Facultad de Medicina, Universidad Nacional de Colombia, Carrera 30 No. 45-03, Edificio 471, 111321 Bogota, Colombia

**Keywords:** CAR-T, Autoimmunity, Lupus, Immunotherapy

## Abstract

Chimeric antigen receptor (CAR) based therapies are promising in systemic autoimmune rheumatic disease (SARD) according to recent case reports. To establish the state of the art of the applications of CAR therapies in rheumatology, PubMed, EMBASE, Lilacs, as well as clinical trial registries were searched. Any type of original article that reported the use of these therapies in patients with SARD was included. Forty-eight studies were included from databases. Ninety-five ongoing trials were included from clinical trials registries. The most used type of CAR was a CAR-T against CD19, showing good preliminary results. Other targets used were BCMA, PD-1, a modified HLA and citrullinated peptides among others. The use of other cell types such as natural killer and regulatory T cells was also found. CAR-based therapies in rheumatology are in an early stage of development but with promising results. There has been a notable growth in human patients treated with this intervention in the last 2 years. This and other innovative designs will offer a wide range of new therapeutic possibilities. Results of ongoing clinical trials are needed to establish their efficacy and safety in SARD.

**Supplementary Information:**

The online version contains supplementary material available at 10.1007/s10238-025-01717-9.

## Introduction

The prevalence of autoimmune diseases is increasing [1, 2]. Systemic rheumatic autoimmune diseases (SARD) comprise rheumatoid arthritis (RA), systemic lupus erythematosus (SLE), systemic sclerosis (SSc), inflammatory myopathies (IM) and Sjögren's syndrome (SS). Impact of these diseases on quality of life, health services utilization and associated costs will significantly grow in the coming years [3]. New therapeutic approaches are necessary to broaden the therapeutic armamentarium for this group of diseases that are frequently refractory to the treatments available today [4].

**Fig. 1:**
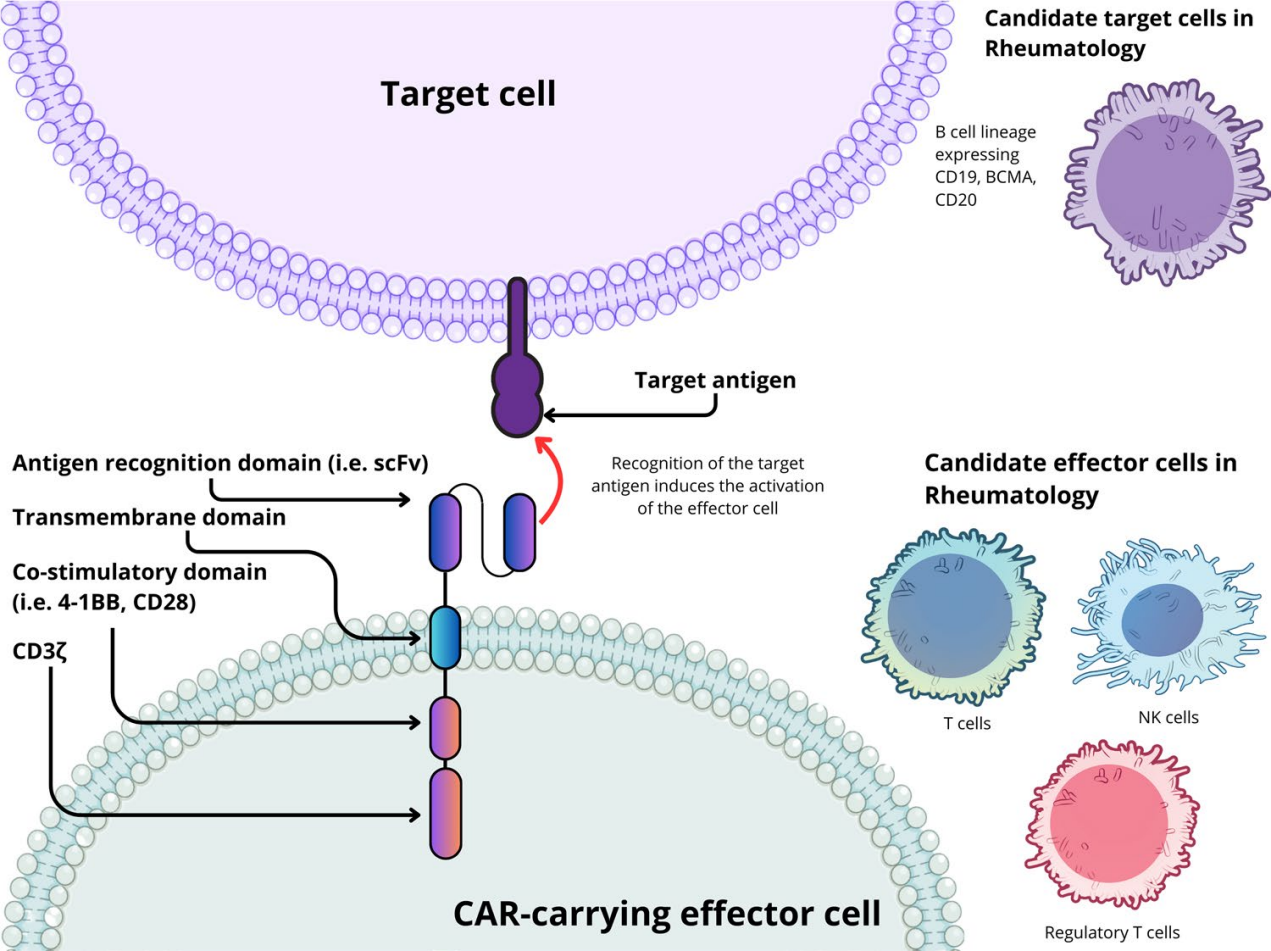
Basic structure and function of CAR therapies used in rheumatology. Different effector cells (i.e., T cells, NK cells and regulatory T cells) have been used for the treatment of SARDs or have been tested in animal models. These effector cells express the chimeric antigen receptor usually via lentiviral transduction. Once expressed, the CAR protein can recognize a target antigen and induce the activation of the carrying cell bypassing MHC restriction or the need for additional signals. Activation of cytotoxic T cells or NK cells triggers the selective elimination of cells expressing the target antigen, while the activation of Tregs induces their suppressive function directed at target cells. *NK cell*: Natural killer cell, *scFv*: single chain variable fragment, *Tregs*: regulatory T cells, *MHC*: Major histocompatibility complex. Illustration created with canva.com with adapted illustrations from NIAID NIH BIOART and Servier Medical Art licensed under CC BY 4.0

Chimeric antigen receptors are fusion proteins created to provide premeditated specificity for T cell activation [5]. They are typically composed of an extracellular antigen-binding domain, an extracellular spacer, a transmembrane domain and an intracellular signaling domain responsible for T cell activation. This composition confers them with the great advantage of modularity [6, 7]. That is, the possibility of exchanging the different domains for others to modify the function of each construct either in terms of recognition or signaling. The cell's effector mechanism can be directed toward a particular target by modifying the extracellular antigen-binding domain. This allows directed triggering of T cell functions while avoiding the limitations normally imposed by conventional T cell activation (Fig. 1).

To date, 6 CAR-T therapies have been approved for the treatment of different B cell malignancies [5, 8, 9]. This successful experience has sparked great interest in the possibility of expanding its applications to other diseases, particularly autoimmune conditions. CAR therapies have initiated their application in humans with different autoimmune pathologies such as SLE, SSc, antisynthetase syndrome and antiphospholipid syndrome [10–15]. The surge in the number of publications on the subject makes it necessary to perform a scoping review to update the outlook of the use of CAR therapies in systemic rheumatic diseases.

## Methods

A search was performed in PubMed, EMBASE and Lilacs databases. Additionally, different clinical trial registries were consulted (see Supplementary Table 1). Search was not restricted by language or publication date. The search strategy can be consulted in Supplement 1. All studies in humans or animal models that used CAR therapy in any of its forms in patients with SLE, RA, SSc, IM, SS, antiphospholipid syndrome (APS) or its corresponding models were included. The last search date was November 21, 2024. Studies that evaluated CAR therapy in organ-specific autoimmunity or autoimmune neurological disorders were excluded.

### Data extraction

The search results were evaluated by title and abstract by 2 researchers (DN and GQ) using the rayyan.ai website tool for reference management. Those considered potentially relevant were obtained for full-text review. Any disagreement was resolved by consensus between both reviewers. The researchers extracted data from the included literature using a pre-established form.

### Data analysis

A descriptive analysis of the data obtained was performed. The information was summarized in tables with the results of the articles included in both the databases and the clinical trial registries.

## Results

**Fig. 2:**
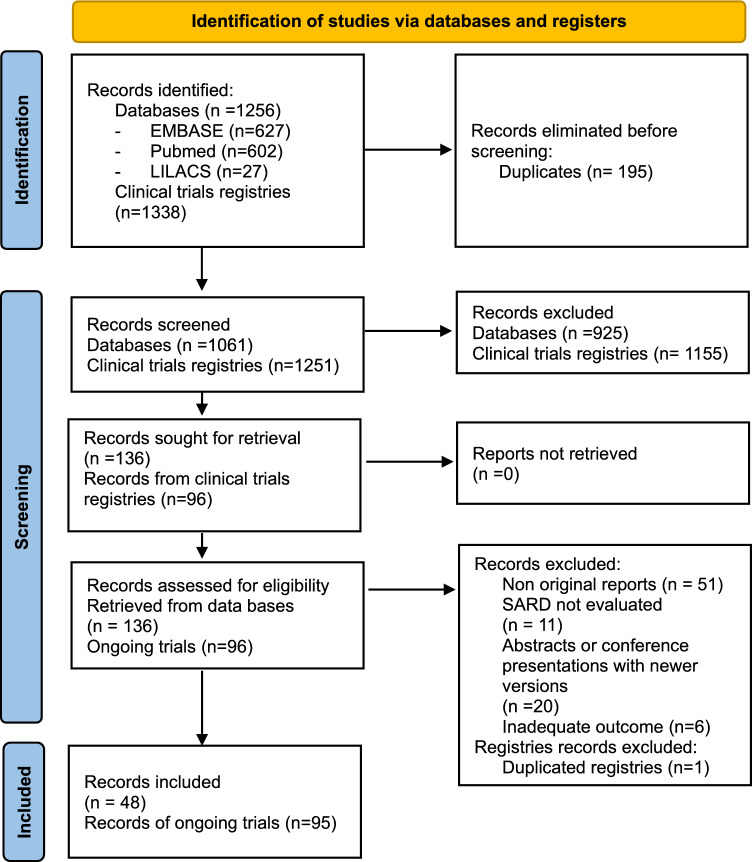
PRISMA flowchart

Searches were conducted in PubMed, EMBASE and LILACS databases. Our search strategy yielded 1256 records. After eliminating 195 duplicates, we screened 1061 records, of which 136 studies were retrieved for full-text evaluation. Of these, forty-eight studies were eligible. Reasons for exclusions are listed in Fig. 2.

### In vitro studies

Four in vitro studies were included. Two of these used CAR-T with CD19 target, both evaluated the activity of CAR-T in cytotoxicity assays demonstrating the specific elimination of CD19+ cells [16, 17]. See Supplementary Table 2.

One study evaluated the use of a “universal CAR-T” directed against an adapter molecule, in this case fluorescein isothiocyanate (FITC, a fluorochrome commonly used in flow cytometry). FITC was used to label citrullinated autoantigens (vimentin, type II collagen, fibrinogen, tenascin-C). This approach allows to direct the action of the CAR-T against different types of autoreactive B cells that recognize those autoantigens by administering specific FITC-labeled citrullinated autoantigens [18]. Using hybridoma cells immunized against those different citrullinated peptides, they demonstrated their specific elimination upon recognition of the appropriate FITC-labeled citrullinated peptide. This type of CAR-T was able to kill B cells isolated from splenocytes of collagen-induced arthritis mice model, using FITC-labeled type II collagen. Researchers verified the elimination of purified B cells from patients with rheumatoid arthritis in the presence of specific FITC-labeled antigenic peptides detected in these patients. It was also shown that the activity of the CAR-T was restricted by the presence of the appropriate FITC-labeled antigenic peptides, being inactive when tested with a FITC-mock peptide.

Meng et al. described the use of an NK cell with a third-generation chimeric autoantibody receptor (CAAR-NK), using the LA peptide as an extracellular recognition domain [19]. This epitope would serve as a cognate antigen for autoreactive B cells to this peptide. Recognition of said epitope by an autoreactive B cell triggers the cytotoxic function of the CAAR bearing T cell (thus functioning as bait). Elimination of 70% of lymphoma cells transfected with anti-La B cell receptor (BCR) was demonstrated, as well as the killing of B cells derived from patients with anti-La+ Sjögren's disease.

### Animal studies (Table 1)

**Table 1:** Studies of CAR applications in murine models of SARD

References	Disease model	CAR Type	Target	Outcomes
[20]	SLE	CAR-T	CD19	Prolongation of survival, decrease in the severity of skin lesions, reduction of spleen weight, decreased severity of glomerulonephritis without changes in serology or proteinuria
[21]	SLE	CAR-T	CD19	Significant increase in survival in both models (NZB/W and MRL-lpr), improvement in proteinuria, spleen weight and skin involvement
[22]	SLE	CAR-NK	PD-1 (using PD-L1 as extracellular domain)	Demonstrated activity against cells that express PD-1. Selective elimination of Tfh. In a humanized SLE model, reduced splenomegaly, decreased CD4 + cell counts, B cell counts, IgG and plasmablasts
[23]	SLE	CAR-T	BCMA (using APRIL as extracellular domain)	Decrease in activated B cells and longer survival
[24]	SARD	CAR-T	CD19	Elimination of CD19 + B cells derived from patients with SARD
[26]	RA	CAR-HLA	T cells autoreactive to HLA DR1	50% reduction in the incidence of arthritis, decreased severity in mice that developed it, reduction of autoantibodies
[27]	SSc	CAR-T	CD19	CAR-T increased mortality, worsened pulmonary fibrosis and increased collagen in the lungs, worsening pulmonary hypertension
[28]	SLE	CAR-Treg	CD19	Increase in IL-10, delayed onset of lymphopenia, reduced anti-dsDNA positivity, reduced inflammatory lesions in the spleen, lung and kidney

*SLE*: Systemic lupus erythematosus; *RA*: Rheumatoid arthritis; *SSc*: Systemic sclerosis; *CAR-Treg*: CAR in regulatory T cells; *CAR-HLA*: CAR modified HLA; *CAR-NK*: CAR in NK cell

Three studies evaluated the use of CAR-T in murine models of SLE [20–23]. Jin et al. [20] and Kansal et al. [21] used CD19 as a target, in MRL-lpr and NZB/W mice lupus models. In both, an increase in survival was found in mice treated with the CAR-T, reaching ages that are markedly unusual for the mice models of lupus. Wilson et al. [23] utilized a CAR-T with an APRIL protein as the extracellular domain, seeking the elimination of BCMA+ cells in the BXSB lupus prone mouse model. They observed a longer survival in the CAR-T-treated mice than in the controls. Peng et al. [24] presented a design of a conventional CAR-T directed against CD19 using a 4-1BB stimulatory domain which has been associated with a lower incidence of CRS and ICANS. In vitro data showed similar potency to other constructs in the killing of Nalm6 CD19+ cells transferred to mice.

Reighard et al. [22, 25] described the use of CAR-NK cells to eliminate T follicular helper cells (Tfh). To achieve this, an extracellular domain composed of PD-L1 was devised, looking for the selective elimination of cells that highly expressed PD-1, such as Tfh cells. This design was used to avoid the targeting of other cells that express lower levels of PD-1 and that would be affected if a conventional extracellular domain of a single chain antibody (scFv) against PD-1 was employed. This, due to the higher affinity of the scFv and cognate antigen interaction, compared to that of PD-1 and PD-L1. This CAR-NK demonstrated the selective elimination of Tfh, sparing other PD-1 positive cell populations. The construct was used in a humanized murine lupus model resulting in a reduction in splenomegaly, CD4+ cell counts, plasmablasts and B cells.

A study in a humanized murine model of collagen-induced arthritis (a model of RA), using DRB1*01:01 transgenic mice, evaluated the feasibility of using modifications to the HLA DR1 structure to induce the elimination of autoreactive cells [26]. The design was based on the replacement of the transmembrane and cytoplasmic domains of HLA DR with CD28 and CD3 zeta domains. This construct was transfected into mice CD8 cells looking to eliminate T cells that recognized the immunodominant peptide of type II collagen presented by the modified HLA DR1. Cytotoxicity tests demonstrated the ability to eliminate type II collagen-specific CD4+ cells. Treated mice showed a 50% reduction in the incidence of arthritis and, in those that developed the disease, a slower onset and lower severity compared to controls. Autoantibody levels were also reduced in the intervention group.

In a SSc murine model, Avouac et al. [27] studied the effect of CD19+ cell depletion. The Fra-2 female model was used in 8 mice treated with a CAR-T against CD19, compared with 16 untreated controls and 15 treated with a monoclonal anti-CD20 antibody. A marked reduction in CD19+ B cells was found in those treated with the CAR-T. Mice that received the CAR-T had an increase in mortality compared to the other groups, with exacerbation of pulmonary fibrosis and higher collagen content in the lungs, as well as worsening of pulmonary hypertension. The possible low influence of autoimmunity in the pathogenesis of the murine model and that the main component of the pulmonary inflammatory infiltrates in this model are T cells were proposed as an explanation for the lack of effectiveness. The possible risk of exacerbation of lung disease with this approach was also of note.

The use of CAR bearing regulatory T cells (CAR-Tregs) has been reported in an animal model of SLE using CD19 as a target in a second-generation CAR joined to Foxp3 gene by a self-cleaving peptide [28]. The overexpression of Foxp3 gene was used to ensure the stability of a Treg phenotype. The authors showed the persistence of the suppressive capacity after the stimulation of the CAR-Tregs. They also reported the antigen specific activation of the CAR-Tregs and robust suppression of autologous B cell proliferation. In a humanized NSG mice model, treatment with the CAR-Treg led to increased levels of IL-10, decreased positivity of anti-dsDNA, delayed the onset of lymphopenia and decreased inflammation in lung, spleen and kidney biopsies.

### Human studies (Table 2)

**Table 2:** Studies of CAR applications in humans

References	Publication Type	Number of patients	Disease	CAR Type	Target	Outcomes
[29]	Case report	1	APS	CAR-T	CD19	Negativization of anticardiolipin IgM, withdrawal of anticoagulation without recurrence of VTE
[12]	Case report	1	SLE	CAR-T	CD19/ BCMA	Disease remission, negativization of ANA, normalization of complement
[34]	Case series	15*	SLE/SSc/IM	CAR-T	CD19	SLE: remission by DORIS, SLEDAI 0, anti-DNA negativization, resolution of proteinuria IM: major ACR/EULAR improvement, normalization of muscle function, resolution of extramuscular activity SSc: decreases in EUSTAR and Rodnan score
[35]	Case series	6**	SSc	CAR-T	CD19	Median decrease in EUSTAR AI of -2.1, median decrease of mRSS -8. Significant decreases of ANA, anti-RNA Pol III and anti-Scl-70 No progression of lung or heart involvement
[36]	Case report	1	SSc	CAR-T	CD19	Improvement in Rodnan score, improvement in dyspnea, pulmonary function tests and extent of interstitial involvement by tomography, decrease of anti-Scl-70
[37]	Phase I trial	13	SLE	CAR-T	CD19/ BCMA	Remission in 9/13, 12/13 at least low disease activity, 11/13 negativization of autoantibodies
[38]	Case report	1	SLE	CAR-T	CD19	Resolution of refractory SLE thrombocytopenia
[39]	Case report	1	SSj	CAR-T	CD19	Negativization of ANA and Ro52, improvement of ILD and sicca symptoms
[40]	Case report	1	RA	CAR-T	CD19	Remission of RA by DAS28 (6 to 1.9) and CDAI (28 to 0). Seroconversion of anticitrullinated protein antibody
[41]	Case report	1	RA	CAR-T	CD19/ CD20	Drug-free remission by DAS28, decreases in rheumatoid factor titer
[42]	Case report	3	IM/SSc	alloCAR-T	CD19	IMNM: Remission by TIS, normal CK, anti-SRP negativization SSc: Improvement by ACR-CRISS. Significant decrease in anti-Scl-70, improvement in lung and cardiac involvement by imaging
[43]	Case report	1	IM	CAR-T	CD19	Improvement in strength by MMT-8, decreases in CK and anti-SRP antibodies
[44]	Case report	1	IM	CAR-T	BCMA	Recovery of muscle function, normalization of CK, improvement in MRI, negativization of anti-SRP
[45]	Retrospective cohort study	58	SLE, RA, SS, others	CAR-T	CD19	Disappearance of autoantibodies and less use of DMARDs
[46]	Case report	1	JDM	CAR-T	CD19	Normal strength, resolution of myositis in MRI, improvement of skin rash, ulcers and calcinosis
[47]	Case report	1	SLE	CAR-T	CD19	Decreased disease activity, improvement in kidney function allowing withdrawal of hemodialysis
[48]	Case report	1	SLE	CAR-T	CD19	Clinical remission and marked histological improvement in a biopsy 2 months after CAR-T infusion
[49]	Case report	2	SLE	CAR-T	CD19	Decrease in SLEDAI from an initial score of 12 for both patients to 0 and 4

^*^Includes patients previously reported in references 31 to 37. ^**^Describes a longer follow-up of SSc patients included in reference 39 and 2 new SSc patients. SLE: Systemic lupus erythematosus; SSc: Systemic sclerosis; IM: Inflammatory myopathy; IMNM: Immune mediated necrotizing myopathy; JDM: Juvenile Dermatomyositis; APS: Antiphospholipid syndrome; SS: Sjogren's syndrome; RA: Rheumatoi arthritis; SARD: Systemic autoimmune rheumatic disease; others: Psoriasis, polymyalgia rheumatica; alloCAR-T: Allogeneic CAR-T; ANA: Antinuclear antibodies; CK: Creatine kinase; VTE: Venous thromboembolism; MMT: Manual muscle testing; TIS: Total improvement score; ESSDAI: Sjogren syndrome disease activity index; ILD: Interstitial lung disease; DMARD: Disease-modifying antirheumatic drug

#### Initial reports

The reports by Schmelz et al. and Zhang et al. [12, 29], in the incidental treatment of autoimmune diseases in patients with B cell malignancy who received a CAR-T (against CD19 and compound CD19/BCMA, respectively), were the first experiences reported in human autoimmunity. The first case described a 67-year-old patient with antiphospholipid syndrome with multiple recurrent episodes of venous thromboembolism who, after therapy with the CD19 CAR-T, achieved seroconversion of anticardiolipin antibodies and withdrawal of anticoagulation without recurrence of thromboembolic events. The second case was a 41-year-old patient with a history of SLE for 20 years, whose level of activity or system involvement was not described, that after receiving treatment with CAR-T presented normalization of complement and negativization of antinuclear antibodies, as well as an inactive state of the disease as described by the authors.

#### The German experience

A group of researchers from the University of Erlangen in Germany [10, 11, 13, 15, 30–33] published several case reports using a CAR-T against CD19 as a compassionate therapy in SLE initially and then extending to systemic sclerosis and inflammatory myopathy. In all cases, the response was favorable. The follow-up of these patients was updated in a series of cases that included 6 additional patients to those presented in previous reports [34]. Follow-up of 15 patients was presented (8 with SLE, 3 with IM and 4 with SSc). All had previous inadequate responses to at least two lines of immunosuppressive treatments including conventional drugs, biologicals, JAK inhibitors or other therapies. SLE patients had a mean SLEDAI-2K of 13, at least a BILAG A score and histologically proven lupus nephritis. Patients with inflammatory myopathy and systemic sclerosis had interstitial lung disease with decreased forced vital capacity and carbon monoxide diffusion. The muscle involvement of patients with myopathy was active with marked elevations of creatine kinase (CK). Skin involvement in systemic sclerosis had a mean modified Rodnan score (mRSS) of 25.5. More than half of the patients had received therapies directed against B cells and/or cyclophosphamide.

The outcomes in all patients were favorable, SLE patients achieved remission according to DORIS criteria at 6 months, with an SLEDAI-2K of zero. The follow-up, the longest being 29 months, showed the absence of activity in the 8 patients. Disappearance of anti-DNA antibodies and proteinuria, as well as the normalization of C3, was also noted. One of the patients presented proteinuria four months after the infusion, a renal biopsy was performed that did not show lupus nephritis but did show podocytopathy, persisting with mild proteinuria at the end of follow-up. Patients with inflammatory myopathy all achieved major clinical response in the total ACR/EULAR improvement score, with normal muscle function and CK levels after 3 months, extramuscular activity also disappeared. Patients with systemic sclerosis showed a decrease in EUSTAR activity indices (median -4.2) and mRSS scores (median-9). All the 15 reported patients accomplished withdrawal of steroids and all immunosuppressants to the last available follow-up. Regarding autoantibodies, 7 SLE patients sustained negativity for anti-DNA and anti-SM antibodies. Ro-60 was positive in 3 patients at the end of follow-up. Antinuclear antibodies decreased but remained positive in almost half of the patients. In the other diseases, a decrease in autoantibodies was found but with a less frequent negative seroconversion, with persistence of autoantibodies such as Jo-1, PL-7, PM-Scl-100 and Scl-70. A longer follow-up of the first 4 SSc patients was published later with the addition of two new patients [35]. Skin involvement improved with a median decrease of 8 points, and the EUSTAR activity index also decreased with a median of 2.1. Autoantibodies, including ANA, anti-RNA Pol III and Scl-70, significantly declined but were not eliminated.

Safety of the therapy was also described, with no moderate or severe cytokine release syndrome (CRS) or immunoeffector cell-associated neurotoxicity syndrome (ICANS) reported, although mild CRS was present in 11 of the 15 patients. Nine patients required tocilizumab for CRS. Several infections were reported, most of them being mild. Only one patient required hospitalization for pneumonia 7 weeks after CAR-T infusion. The B cells reconstituted at 4 and 12 months showed a *naïve phenotype*, without evidence of class switching, which supports the possibility of “resetting” the development of autoimmunity.

In addition to these patients, Merkt et al. [36] described the compassionate use of a CD19 CAR-T in a 38-year-old patient with Scl-70+ systemic sclerosis with pulmonary involvement by progressive non-specific interstitial pneumonia (NSIP). The patient had been refractory to cyclophosphamide, mycophenolate and nintedanib. After the infusion of a third-generation CAR-T against CD19 with CD28 and 4-1BB stimulatory domains, the patient presented gradual remission of dyspnea, regression of skin involvement and puffy fingers improved. High resolution chest tomography images showed a drastic improvement in the extent of NSIP. Regarding the safety of the therapy, a CRS grade 1 was reported.

#### The first phase I trial of CAR-T in Lupus

An open label, single-arm, phase 1 clinical trial using a compound CAR-T, consisting of independent functioning CARs that were directed against CD19 and BCMA [37]. These targets would allow broad depletion of B cells and plasma cells. Thirteen patients with SLE (mean SLEDAI-2K of 10.6) and lupus nephritis (class III, IV or V) were included, 2 of them with comorbid diffuse large B cell lymphoma (DLBCL). They had received at least 2 previous lines of treatment for SLE. Nine patients achieved remission, while three others had low disease activity. All but one patient showed seroconversion of autoantibodies. One of the patients that achieved remission suffered a relapse 4 months later, then received another dose of the CAR-T treatment attaining remission for 6 months with a subsequent relapse requiring pharmacological treatment. Concerning safety, all patients experienced CRS but none of them suffered ICANS. There were few reported infections, all of them mild. Nevertheless, most of the patients required immunoglobulin therapy for hypogammaglobulinemia.

#### Other experiences

Other case reports have described the effectiveness of B cell targeted CAR-Ts for different manifestations of SARD. Li et al. [38] described the resolution of refractory thrombocytopenia in a SLE patient. Sheng et al. [39] reported the incidental use of a CAR-T therapy against CD19 in Sjögren's disease in a 76-year-old patient with lymphoma. The patient had a 10-year history of anti-Ro+ Sjögren's disease, with interstitial lung disease. After the infusion of CAR-T therapy, she presented grade 1 CRS and a potential infection that required antibiotic treatment. She also suffered a decompensation of heart failure. On day 90 after the infusion, antinuclear antibodies and Ro52 turned negative, interstitial lung disease, sicca symptoms improved and the ESSDAI score decreased from 4 to 2. One patient with myasthenia gravis (MG) and comorbid rheumatoid arthritis received a CD19 CAR-T indicated primarily for the former diagnosis [40]. She had been previously treated with multiple therapies for myasthenia, including thymectomy, acetylcholinesterase inhibitors, glucocorticoids, azathioprine, intravenous immunoglobulins, rituximab and eculizumab. She had not received specific treatment for her rheumatoid arthritis, despite high activity disease (DAS28 6, CDAI 28), fearing adverse effects of combination treatments. After the administration of the CAR-T, she achieved remission of both MG and RA with seroconversion of anticitrullinated protein antibody (ACPA). Another patient with RA, previously treated with methotrexate, hydroxychloroquine and tocilizumab with persistent low to moderate activity, received a CD19-CD20-directed CAR-T indicated for a relapsed DLBCL attaining RA drug-free remission [41].

Wang et al. [42] used allogenic CD19 CAR-T cells derived from healthy donors T cells. These cells were transduced with a lentivirus coding the CAR construct and then edited by CRISPR-Cas9 to knock out human leukocyte antigen (HLA)-A, HLA-B, class II major histocompatibility complex transactivator (CIITA), T cell receptor alpha constant (TRAC) and PD-1. These modifications render these CAR-T cells compatible for allogeneic therapy. One patient with immune mediated necrotizing myopathy (IMNM) and two patients with SSc were treated with these engineered allogeneic CAR-T cells. The patient with IMNM achieved remission with negative autoantibodies (previously anti-SRP+). Both patients with SSc showed clinical improvement by the American College of Rheumatology Composite Response Index in Systemic Sclerosis (ACR-CRISS). Anti-Scl-70 antibodies also decreased significantly.

Volkov et al. [43] reported the case of a patient with IMNM anti-SRP positive that was treated with a CD19 CAR-T, with improvement in strength (by MMT-8 score), and decreases in CK and anti-SRP antibodies levels. Another case was described by Qin et al. [44]. In this case, the CAR-T was directed against BCMA in a highly refractory patient who was bedridden prior to treatment. After the intervention, he recovered the ability to walk; after nine months, the neurological examination was nearly normal with only proximal residual weakness in the lower limbs. The MMT-8 muscle test score increased from 96 to 137, CK normalized and MRI showed reduced activity in the arms and thighs. The improvement was sustained without the need for additional treatments for 18 months. Grade 1 CRS occurred, with no other adverse effects.

Utilizing real-life data from 90 million patients in the US, Wang et al. [45] analyzed patients who had received any of the CAR-T therapies approved in the US for the treatment of non-Hodgkin lymphoma and who had a comorbid rheumatic disease. Using propensity score matching, they evaluated the outcomes of 58 patients with rheumatic disease (24 with RA, 10 with SLE, 5 with SS) and other diseases such as polymyalgia rheumatica, ankylosing spondylitis and psoriasis (16 other patients), with 58 patients without rheumatological disease. No differences were found regarding toxicity, time to next treatment for their malignancy or mortality. After receiving the CAR-T in patients with rheumatic disease, of 15 patients who had positive antinuclear antibodies, only 5 remained positive; the 2 patients with positive anti-DNA became negative; of the 11 patients with positive rheumatoid factor at baseline, only 1 persisted positive. Steroid use went from 50% of patients with rheumatic disease to 31%. The use of methotrexate decreased from 17 patients to 4 and that of hydroxychloroquine from 10 to 1 patient. This illustrates a possible benefit of the therapy in their respective autoimmune diseases.

#### Reports in pediatric patients

Several case reports have described the use of CD19 CAR-T for pediatric patients with SARD. Nicolai et al. [46] reported the first patient with juvenile dermatomyositis in a 12-year-old with a highly active disease, refractory to previous treatments that included cyclophosphamide, intravenous immunoglobulin and mycophenolate. CAR-T treatment prompted marked improvement, with resolution of the severe skin rash and regaining normal strength. Krickau et al. [47] described the first pediatric SLE patient with severe manifestations including class IV lupus nephritis requiring hemodialysis despite previous treatment with mycophenolate, belimumab and cyclophosphamide. CAR-T therapy led to rapid diminishing of disease activity and partial renal response (because of decreased but persistent proteinuria) with sufficient recovery of kidney function to permit hemodialysis withdrawal. De Benedetti et al. [48] reported a 16-year-old SLE patient with lupus nephritis class II and V, interstitial lung disease and pulmonary hypertension that, after CAR-T administration, accomplished drug-free remission with marked improvements in renal histopathology and pulmonary hypertension. Later, Xue et al. [49] described two additional pediatric SLE patients, who were refractory to previous treatments and had severe disease activity with baseline SLEDAI scores of 12 for both patients. After CAR-T infusion, the improvement was notable, with reductions of the SLEDAI score to 0 and 4. All these patients developed mild CRS, but none suffered ICANS or other severe adverse events.

### Experiences reported in abstracts (Supplementary Table 3)

Schett et al. [50] presented preliminary data of the first eight patients enrolled in a phase I/II study of a CD19 CAR-T for the treatment of B cell-mediated autoimmune disease (SLE, IM, SSc). Five of these patients had efficacy data available, showing that 3 out of 3 SLE patients achieved DORIS remission, 1 of 1 IM patient major response in ACR improvement score and 1 of 1 patient with SSc had stable lung function. All these patients stopped medications after CAR-T infusion. CRS was absent or mild, and no cases of ICANS were reported. Similarly, Cortes-Hernandez et al. [51] reported preliminary results of a phase I/II trial of YTB323 a rapidly manufactured CD19 CAR-T for the treatment of refractory SLE, 3 patients with efficacy data showed reductions in SLEDAI scores and improvement in biomarkers. Of the six patients with safety data 4 presented with CRS grade 1 or 2 and none developed ICANS, one patient developed pneumonia. Hu et al. [52] presented preliminary safety and efficacy data of three patients treated with another CD19 CAR-T, with decreases of baseline SLEDAI scores from 8 to 14 to 0 or 1 after the infusion. Podoll et al. [53] presented two patients with lupus nephritis recruited in an ongoing clinical trial, both patients showed reductions in urine to creatine ratios and SLEDAI scores, although with limited follow-up (56-and 28-day post-infusion).

Some other experiences have been preliminarily reported in abstracts using different types of CARs and targets. Use of CAR-Tregs in RA has been described in cell studies [54, 55]. In these, citrullinated vimentin was used as a target to direct the activation of the CAR-Tregs. Specific activation by the citrullinated protein and not by the unmodified vimentin was demonstrated in vitro. Activation of these cells suppressed effector CD4 and CD8 cells in the presence of citrullinated vimentin. When cultured with synovial fluid from patients with RA, *in vitro* activation and proliferation of CAR-Tregs were also found.

Park et al. [56] studied the targeting of CAR-Ts against profibrotic macrophages using a CAR-T against CD206. In a murine model of systemic sclerosis of C57BL/6 mice injected with bleomycin. Elimination of CD206+ macrophages was found, as well as a decrease in dermal thickness and the reduction of genes associated with disease progression in skin and lung tissues.

Preliminary results of phase I studies with a dual CAR-T against CD19/BCMA have been reported for the treatment of SLE and systemic sclerosis refractory to standard treatments [57, 58]. In the case of systemic sclerosis, 3 treated patients were reported, one of them with interstitial lung disease as the main manifestation. After the administration of the CAR-T, improvement in cough and the extent of the disease was shown by tomography, with a 9-month follow-up without requiring other additional treatments. The other two patients received treatment without complications, but no other outcomes were reported. Regarding its application in SLE, 12 patients with severe disease activity without response to multiple treatments received CAR-T therapy. All patients presented CRS grade 1 after infusion, none of them developed ICANS. Infections occurred in four patients and were mild. SLEDAI-2K decreased from a mean of 18.5 to 1.5, with persistence of low-grade proteinuria in some patients that was attributed to established damage. All patients achieved low disease activity and were able to discontinue all SLE medications. No disease exacerbations were reported during follow-up.

Rimann et al. [59] showed the feasibility of creating a CAR-T with the possibility of controlling its cytotoxic capacity by directing its antigen-binding domain against an anti-CD19 Fab fraction that was used as a “switchable CAR-T” (sCAR-T). This way, it was possible to control the activity of sCAR-T, which would exert its cytotoxicity only in the presence of the anti-CD19 switch. This design was tested in BXSB mice (a murine model of SLE), showing that treatment with the sCAR-T and administration of the switch led to decreases in B cells, autoantibodies and nephritis. Longer survival of the treated mice was also found.

### Clinical trial records

**Fig. 3:**
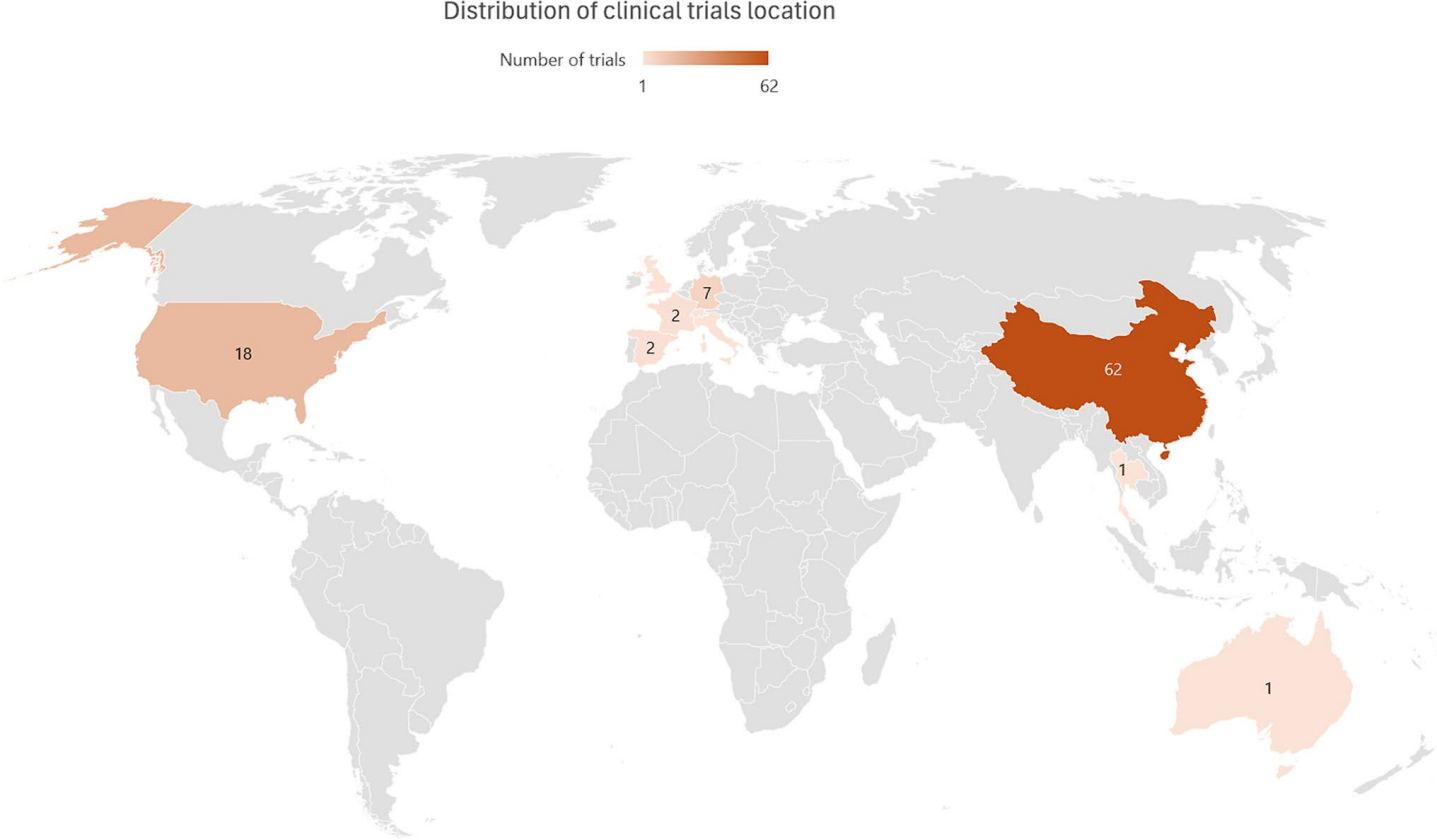
Distribution of clinicaltrials.gov registered trials

A search was carried out in the clinical trial registries listed in Supplementary Table 3. In total, 1338 trials were obtained, of which 95 were included, distributed as follows: 90 in clinicaltrials.gov, 4 in chiCTR and 1 study in drks.of. The identified studies are summarized in Supplementary Table 4. The locations of the trials in clinicaltrials.org are shown in Fig. 3.

Regarding the status of the 95 trials, 68 were in the recruitment phase, 25 had not yet started recruitment and 2 had an unknown status. Most studies are phase I studies (58 of 95), 20 studies are phase I/II and three studies are phase II. Fourteen studies did not report which phase they corresponded.

Almost half of these trials will study conventional CAR-T directed against CD19 (47/95). Eighteen of these studies will evaluate a compound CAR-T against BCMA and CD19, being the second most common type of target under study. Other conventional CAR-T targets include CD19/CD20, CD20/BCMA, BAFF and CD7. Apart from CD7, which seeks to eliminate T cells, all these therapies are directed toward the depletion of the B cell lineage.

Other types of CAR therapies included 11 studies that are evaluating the use of a CAR-NK, of these, one will study a CAR-NK with switch (sCAR-NK). All these 11 studies use CD19 as a target. Besides this, other novel designs include a CAR-T that uses mRNA as an expression method for the construct, thereby avoiding genomic integration and consequently the unwanted persistence of the CAR. Two studies will evaluate a 4SCAR-T, a fourth-generation CAR-T directed at multiple targets (CD19, BCMA, CD138 and BAFF-R). Two studies will evaluate the use of allogenic CAR-T cells, directed against CD19.

## Discussion

The treatment of these diseases is an unmet need. Currently available treatments are mainly based on non-specific immunosuppression, with the consequent lack of response in a substantial proportion of patients and the adverse effects of therapies with broad effects. In addition, long-term therapies are often required to prevent relapses of the disease [60].

The use of CAR-based therapies has been rapidly growing in the last five years. The therapy with the most experience is the use of CD19 CAR-Ts for the depletion of B cells that express said marker. B cells have been frequently implicated in the pathogenesis of different autoimmune diseases. Their role in the synthesis of autoantibodies, inflammatory cytokines and antigen presentation [33, 61, 62] makes them one of the most common therapeutic targets in different autoimmune diseases. However, the effectiveness of rituximab, the main strategy in use for depletion of these cells, has frequently been disappointing. Incomplete depletion of B cells in tissues and lack of CD20 expression in plasmablasts and long-lived plasma cells have been proposed as mechanisms for the lack of effectiveness of rituximab in many patients [5, 63, 64]. Anti-CD19 CAR-Ts can achieve deeper depletion of B cells, both due to targeting a more widely expressed antigen in the B cell lineage and their ability to access tissues. This approach has been tested in autoimmunity with preliminary evidence supporting its usefulness and safety mainly in SLE, SSc and IM, and more scantly in RA, SS and antiphospholipid syndrome. Most of the ongoing clinical studies in autoimmune disease are studying this design in SLE, SSc and IM, which are diseases urgently in need for effective therapies.

The use of anti-BCMA CAR-T has also been tested in humans in diseases such as SLE and necrotizing myopathy, in compound designs with CD19 or as a stand-alone target, also showing promising results. The use of anti-CD19 and BCMA composite CAR-T constructs is the second most studied therapy in phase I and II clinical trials.

Profound B cell depletion has been proposed to “reset” autoimmunity, basing its effectiveness on the elimination of the B cells responsible for the production of autoantibodies relevant to the pathogenesis of these diseases. Furthermore, the reconstitution of B cells in patients treated with this type of therapy occurs with naive B cells. This would explain the lack of disease recurrence even though patients experience reconstitution of this lineage [33].

Results of the phase I and II studies are needed to corroborate the safety and strengthen the evidence of its usefulness, which must be confirmed in future studies because no published study has been compared against a control intervention. However, the striking improvements in patients highly refractory to standard therapies that received compassionate treatments and the results of the first phase I trial of a BCMA/CD19 CAR-T suggest that this design has a high probability of proving effective.

Anti-CD19 CAR-T therapies have accumulated great experience for the treatment of lymphomas. In patients treated for this indication, the main long-term complications are known to be persistent B cell depletion (25–38%), hypogammaglobulinemia (18–74%), cytopenia (3–17%) and late infections (9–61%) [65]. These events were for the most part minor. The incidence of complications such as CRS and ICANS appears to be lower in SARD patients than in patients treated for hematological malignancy. This is probably due to a lower target burden in patients with rheumatologic disease and no malignancy. Another potential adverse effect that has recently raised concern is the development of T cell malignancy. Twenty-two cases of this malignancy have been reported as of December 31, 2023. In 3 of these cases, the presence of the CAR transgene was detected in the malignant clones. However, it should be noted that more than 27,000 CAR-T therapies have been administered. Put into perspective the overall risk of developing T cell cancer appears to be low [66]. This finding highlights the need for long follow-up times to have a clearer picture about the safety of these therapies.

One of the future directions of these treatments is to simplify the manufacturing process. The use of allogeneic cells ready for application as an “off the shelf” treatment has been studied with NK cells, which are advantageous because of the elimination of the risk of graft versus host reactions and the ease of obtaining them leading to lower costs [6, 67]. The disadvantages of these cells lie in their short survival, absence of a memory phenotype, reduced in vivo* expansion* and weak cytotoxicity.

Patient selection for these therapies will be a challenge, owing to the high cost inherent to this highly personalized treatment and the risk of adverse effects. The use of current CAR-T cell therapies will probably be reserved for patients refractory to multiple standard therapies and with highly active, life-threatening disease activity, as is the case of the patients eligible for ongoing phase I/II studies. Also, the development of academic point of care CAR-T manufacturing can significantly decrease the cost of these therapies, improving accessibility [68].

Other therapies using innovative CAR modalities are still in an early stage of development but promise to further expand therapeutic possibilities. One of the most relevant is the modification of HLA molecules associated with disease. This pursues the elimination of autoreactive cells that recognize antigenic peptides presented by said modified HLA. However, the complexity of the construct, the apparent low affinity for the target cells and the absence of proliferation of the bearer cell would be some of the disadvantages of these designs. The so-called universal constructs allow the elimination of several types of autoantibody-producing B cells. Such an approach would probably be limited by the need for the presence of B cells reactive to known antigens and by the uncertainty regarding their pathogenic relevance.

CARs are a possible answer to the limitations of Treg based therapies [69]. CAR-Treg cells are attractive from a safety standpoint. However, the choice of an appropriate target is complicated and could be limited by the localization of the effect in a specific tissue or cell-type. Nevertheless, use of CD19 directed CAR-Tregs could offer a safer alternative while retaining the efficacy of conventional CD19 CAR-Ts in the B cell-mediated autoimmune disease as shown in preclinical models.

## Conclusions

The use of CAR-based therapies is promising for the treatment of systemic autoimmune diseases. This review identified that this therapy has been explored mainly in the use of cytotoxic strategies for B cell depletion. This approach has shown very encouraging preliminary results, with a notable growth in human patients treated with this intervention and in the number of ongoing clinical trials in the last year. Nevertheless, this is just a sample of the universe of possibilities that the CAR technology allows. The use of cells other than conventional T cells, such as NK cells or regulatory T cells, and other innovative strategies studied in in vitro and animal models hold promise to offer new alternatives in the treatment of autoimmune rheumatic diseases.

## Supplementary Information

Below is the link to the electronic supplementary material.

Supplementary file1 (DOCX 246 KB)

## Data Availability

No datasets were generated or analyzed during the current study.
