# Supplementary material for: From myth to bedside: a scoping review of the applications of the chimeric antigen receptor in rheumatology
Source: Clin Exp Med. 2025 Jun 6;25(1):189. doi: 10.1007/s10238-025-01717-9 (PMC12144057; doi:10.1007/s10238-025-01717-9)
Supplement: Supplementary file 1 — Supplementary file1 (DOCX 246 KB) [file 10238_2025_1717_MOESM1_ESM.docx]

**Supplementary material**

1. **Search strategies**

**Pubmed**

("receptors, chimeric antigen"[ MeSH Terms] OR "CD19-specific chimeric antigen receptor"[Supplementary Concept] OR "car t"[All Fields] OR "CAR Treg"[All Fields] OR "Chimeric antigen receptor"[All Fields] OR "CAAR-T"[All Fields]) AND ("autoimmune"[All Fields] OR "autoimmunity"[ MeSH Terms] OR "autoimmunity"[All Fields] OR " autoimmunities "[All Fields] OR "autoimmunization" [All Fields] OR " autoimmunizing "[All Fields] OR "autoimmune disease"[All Fields] OR ("lupus vulgaris"[ MeSH Terms] OR ("lupus"[All Fields] AND "vulgaris"[All Fields]) OR "lupus vulgaris"[All Fields] OR "lupus"[All Fields] OR "lupus erythematosus, systemic"[ MeSH Terms] OR ("lupus"[All Fields] AND "erythematosus"[All Fields] AND "systemic"[All Fields]) OR "systemic lupus erythematosus"[All Fields]) OR ("scleroderma, systemic"[ MeSH Terms] OR ("scleroderma"[All Fields] AND "systemic"[All Fields]) OR "systemic scleroderma"[All Fields] OR ("systemic"[All Fields] AND "sclerosis"[All Fields]) OR "systemic sclerosis"[All Fields]) OR ("scleroderma, systemic"[ MeSH Terms] OR ("scleroderma"[All Fields] AND "systemic"[All Fields]) OR "systemic scleroderma"[All Fields] OR "scleroderma"[All Fields] OR "scleroderma, localized"[ MeSH Terms] OR ("scleroderma"[All Fields] AND "localized"[ All Fields]) OR "localized scleroderma"[All Fields] OR "sclerodermas"[All Fields]) OR ("dermatomyositis"[ MeSH Terms] OR "dermatomyositis"[All Fields]) OR ("polymyositis"[ MeSH Terms] OR "polymyositis"[All Fields]) OR ("arthritis, rheumatoid"[ MeSH Terms] OR ("arthritis"[All Fields] AND "rheumatoid"[All Fields]) OR "rheumatoid arthritis"[All Fields] OR ("rheumatoid "[All Fields] AND "arthritis"[All Fields])) OR (("arthritis"[ MeSH Terms] OR "arthritis"[All Fields] OR " arthritis " [All Fields] OR " polyarthritis "[All Fields]) AND ("experimental"[All Fields] OR "experimentally"[All Fields] OR " experimentals "[All Fields] OR "experimentation"[All Fields] OR "experiments"[All Fields] OR "experimenter"[All Fields] OR "experimenter s"[All Fields] OR "experimenters"[All Fields])) OR ("lupus nephritis"[ MeSH Terms] OR ("lupus"[All Fields] AND "nephritis"[All Fields]) OR "lupus nephritis "[All Fields]) OR "anti-synthetase"[All Fields])

**EMBASE**

('lupus erythematosus'/exp OR 'lupus erythematosus' OR 'rheumatoid arthritis'/exp OR 'rheumatoid arthritis' OR 'myositis'/exp OR 'myositis' OR 'dermatomyositis'/exp OR 'dermatomyositis' OR 'polymyositis'/exp OR 'polymyositis' OR 'systemic sclerosis'/exp OR 'systemic sclerosis' OR 'experimental arthritis'/exp OR 'experimental arthritis' OR 'lupus erythematosus nephritis'/exp OR 'lupus erythematosus nephritis') AND ('chimeric antigen receptor' /exp OR 'chimeric antigen receptor' OR 'chimeric antigen receptor immunotherapy'/exp OR 'chimeric antigen receptor immunotherapy' OR 'chimeric antigen receptor t-cell'/exp OR 'chimeric antigen receptor t-cell')

**LILACS**

(autoimmunity OR "autoimmune disease" OR lupus OR "systemic sclerosis" OR scleroderma OR Dermatomyositis OR Polymyositis OR "rheumatoid arthritis" OR (arthritis AND experimental) OR "Lupus Nephritis" OR "anti-synthetase" OR "artritis reumatoide" OR "esclerosis sistemica" OR "polimiositis" OR "dermatopolimiositis" OR "miopatia" OR "anti-sintetasa" OR "antisintetasa”) AND ("CAR-T" OR "CAR T" OR "chimeric antigen receptor" OR "CAAR-T" OR "CAAR T" OR "CAR-Treg" OR "CAR Treg" OR 'macrophage' OR 'NK' OR 'natural killer' OR 'dendritic cells')

**Supplementary table 1. Records of included clinical trials**

| Record | Website | Country |
| --- | --- | --- |
| US National Library of Medicine | clinicaltrials.gov | USA |
| Australian New Zealand Clinical Trials Registry (ANZCTR) | anzctr.org.au | Australia |
| Brazilian Clinical Trials Registry ( ReBec ) | ensaiosclinicos.gov.br | Brazil |
| Chinese Clinical Trial Registry ( ChiCTR ) | chictr.org.cn | China |
| Clinical Research Information Service ( CRiS ), Republic of Korea. | cris.nih.go.kr | Korea |
| Clinical Trials Registry - India (CTRI) | ctri.nic.in/ Clinicaltrials / advsearch.php | India |
| Cuban Public Registry of Clinical Trials ( RPCEC) | registrarclinico.sld.cu/en/home | Cuba |
| EU Clinical Trials Register (EU-CTR) | clinicaltrialsregister.eu | European Union |
| German Clinical Trials Register (DRKS) | drks.de / drks_web / | Germany |
| Iranian Registry of Clinical Trials (IRCT) | irct.ir | Iran |
| International Standard Randomized Controlled Trial | isrctn.com/ | International |
| Japan Primary Registries Network (JPRN) | rctportal.niph.go.jp | Japan |
| Lebanese Clinical Trials Registry (LBCTR) | https://lbctr.moph.gov.lb/ | Lebanon |
| Thai Clinical Trials Registry (TCTR) | <http://www.clinicaltrials.in.th/> | Thailand |
| The Netherlands National Trial Register (NTR) | trialregister.nl/ | Netherlands​ |
| Pan African Clinical Trial Registry (PACTR) | pactr.samrc.ac.za/ | Africa |
| Peruvian Clinical Trial Registry (REPEC) | clinicalessaysrepec.ins.gob.pe/ | Peru |
| Sri Lanka Clinical Trials Registry (SLCTR) | https://slctr.lk | Sri Lanka |

**Supplementary table 4.**
